# Detecting representative data and generating synthetic samples to improve learning accuracy with imbalanced data sets

**DOI:** 10.1371/journal.pone.0181853

**Published:** 2017-08-03

**Authors:** Der-Chiang Li, Susan C. Hu, Liang-Sian Lin, Chun-Wu Yeh

**Affiliations:** 1 Department of Industrial and Information Management, College of Management, National Cheng Kung University, Tainan City, Taiwan, R.O.C; 2 Department of Public Health, College of Medicine, National Cheng Kung University, Tainan City, Taiwan, R.O.C; 3 Information and Communications Research Laboratories, Industrial Technology Research Institute, Hsinchu, Taiwan, R.O.C; 4 Department of Information Management, College of Information Technology, Kun Shan University, Yongkang Dist., Tainan City, Taiwan; Tianjin University, CHINA

## Abstract

It is difficult for learning models to achieve high classification performances with imbalanced data sets, because with imbalanced data sets, when one of the classes is much larger than the others, most machine learning and data mining classifiers are overly influenced by the larger classes and ignore the smaller ones. As a result, the classification algorithms often have poor learning performances due to slow convergence in the smaller classes. To balance such data sets, this paper presents a strategy that involves reducing the sizes of the majority data and generating synthetic samples for the minority data. In the reducing operation, we use the box-and-whisker plot approach to exclude outliers and the Mega-Trend-Diffusion method to find representative data from the majority data. To generate the synthetic samples, we propose a counterintuitive hypothesis to find the distributed shape of the minority data, and then produce samples according to this distribution. Four real datasets were used to examine the performance of the proposed approach. We used paired t-tests to compare the Accuracy, G-mean, and F-measure scores of the proposed data pre-processing (PPDP) method merging in the D3C method (PPDP+D3C) with those of the one-sided selection (OSS), the well-known SMOTEBoost (SB) study, and the normal distribution-based oversampling (NDO) approach, and the proposed data pre-processing (PPDP) method. The results indicate that the classification performance of the proposed approach is better than that of above-mentioned methods.

## 1. Introduction

Imbalanced data set problems are the issue in the real world and present challenges to both academics and practitioners. It should be noted that the imbalanced dataset is quite common in medical fields due to the imbalance of their class labels. In addition, the high risk/target patients tend to appear in the minority class of the medical dataset. The risk/cost of miss-classification in the minority class is much higher than that in the majority class in medical fields. Most existing classification methods do not have the required qualities in the performance of classification especially when the dataset is extremely imbalanced. For example, Murphey et al. [1], Cohen et al. [2], Sun et al. [3], Sun et al. [4], Li et al. [5, 6], Song et al. [7], Wang et al. [8], and Zou et al. [9] have shown that when limited training data are available, the small size of the minority data will significantly affect the accuracy of medical diagnoses. With imbalanced datasets, when some classes are much larger than the others, most machine learning and data mining classifiers are overly influenced by the larger classes and ignore the smaller ones. As a result, the classification algorithms often exhibit poor learning performances due to slow convergence in the minority classes [3, 4, 10, 11].

A number of solutions for dealing with class imbalance problems have been proposed to handle classification problems in various fields. These approaches can be divided into two types. One creates new algorithms or modifies existing algorithms; example of this type can be found in Hong et al. [11], Peng and King [12], Nguwi and Cho [13], and Lo et al. [14]. For certain types of data sets, this approach can be highly effective for specific classifiers, but the performance of those classifiers is still less than optimal with data sets that have varied characteristics because it is usually difficult to transform the modification procedures from one algorithm to another. The other type of approach in the literature utilizes sampling techniques; these include undersampling and oversampling to adjust the sizes of data to balance the data sets [2–5, 15–18]. The undersampling method reduces the size of data by eliminating samples from the majority class, thus decreasing its degree of influence. However, eliminating data raises the risk of partially removing the complete characteristics that may be represented in the majority class samples. Researchers have discussed various undersampling methods such as the random and directed approaches. These approaches include Kubat and Matwin [19] presented a method called one-sided selection (OSS) that randomly eliminates examples from majority class data sets until the amount of data for the majority class is equal to that of the minority class. Yen and Lee [20] proposed a cluster-based undersampling approach to select representative examples from the majority data to avoid the loss of crucial information.

As for undersampling approach, this study differs from other approaches that randomly draw data from the majority data, raising the probability of imprecisely characterizing the majority data due to the increased influence of noise or outliers in the samples set [21, 22]. Therefore, we propose a systematic procedure using the box-and-whisker plot approach to exclude outliers and the Mega-Trend-Diffusion (MTD) method proposed by Li et al. [23] to construct the distribution of the majority data. The MTD which is a data expansion method used in this study is to reasonably evaluate the domain range of the observed data. Within the estimated domain range, it includes both the reasonable/fitting data and the outliers. MTD is used to construct the membership function of the observed data and to calculate the membership degree of them. The smaller value the membership degree of the data, the more likely an outlier. This study uses theα-cut based on the MTD method to keep the suitable data and to eliminate the outliers. Further, under the estimated distribution of data, this paper takes representative samples from the majority data by settingα-cut values, providing a suitable value forα-cut to determine an appropriate amount of the majority data.

With regard to oversampling, direct resampling is a widely used strategy to balance a class distribution by duplicating minority class examples. Many researchers have adopted oversampling techniques such as those described in Piras and Giacinto [24], Xie and Qiu [21], Tahir [22], and Fernández-Navarro et al. [25]. However, these approaches may suffer from the overfitting problem. In Chawla et al. [26], rather than duplicate examples from a data set, the authors proposed the synthetic minority oversampling technique (SMOTE) to generate synthetic samples in a feature space. Many subsequent studies such as AdaBoost [27] and SMOTEBoost (SB) [28] have adopted this method, and all have confirmed the effectiveness of this approach with regard to enhancing the classification accuracy of minority class data. Unfortunately, these oversampling methods focus on resampling from rare minority class data. Therefore, when the ratio of the minority data to the overall samples is decreasing, the resampling will be too conservative to behave realistically with imbalanced data sets.

Other oversampling methods consider the underlying minority class data distributions. For instance, working in a feature space, Zhang and Wang [29] proposed a normal distribution-based oversampling (NDO) approach to generate normal-synthetic samples with characteristics that are close to those of the raw minority class data with regard to the expected mean and variance. However, with the imbalanced data sets, when there are very few data in the minority class, it is difficult to know whether the data follow a normal distribution.

Therefore, in this paper, based on a two-parameter Weibull distribution, we propose a new oversampling method for generating representative synthetic samples to extend the minority class data. One reason for this is that the distribution used can appropriately characterize the shape of a data set through various shape parameters of the density function [30–32]. Consequently, the method presented in this work is more flexible with regard to the shape of small data sets. Moreover, in our approach, a uniquely counterintuitive hypothesis-testing procedure is constructed to evaluate the shape parameter of the Weibull distribution by choosing the maximal *p*-value of a small data size.

This paper uses four real data sets, Wisconsin Diagnostic Breast Cancer (WDBC) and Parkinson's Disease (PD), Vertebral Column (VC) with two categories: normal and abnormal, and Haberman's Survival (HS), to illustrate the performance of the proposed method. Although accuracy is an appropriate criterion for measuring classification performance, it is not adequate for imbalanced data sets due to the impact of the minority class. As a result, the three criteria including Accuracy (ACC), Geometric Mean (G-mean), and F-measure (F1) are recommended to measure the performance of learning with imbalanced data sets [33]. For the learning tool, we tested the support vector machine (SVM) with a linear kernel function (SVM-linear), Naïve Bayes (NB), *k*-nearest neighbor (KNN), and another type of SVM with a polynomial kernel function (SVM-poly). The experiments show that the SVM with the polynomial kernel function has the best classification performance for raw imbalanced data sets; thus, it is chosen as the learning tool in the subsequent performance comparison among the OSS method, the SB method, the NDO method, the proposed data pre-processing (PPDP) method, the D3C method, and PPDP+D3C method. The D3C is a new hybrid model which combines the ensemble pruning based on k-means clustering and dynamic selection and circulating combination. The D3C model was proposed by Lin et al. [34] to improve the learning of imbalance dataset. It is noted that our proposed method mainly focuses on data pre-processing and the D3C is an ensemble method. Hence, the study proposes the concept of combination of PPDP with LibD3C (PPDP+D3C), that is, the imbalanced datasets are pre-processed by PPDP+D3C, and then are trained by D3C method. The four classifiers set in D3C includes NB, KNN(K = 3), SVM-linear, and SVM-poly. The results show that the combination of PPDP with LibD3C (PPDP+D3C) method has the best classification performance for imbalanced data sets.

The remainder of this paper is organized as follows: Section 2 reviews the literature on the related criteria for evaluating classification performance, the box-and-whisker plot method, and the MTD method. Section 3 introduces the detailed procedure of the proposed method. In Section 4, we present the four real data sets and the detailed experiment methodology, and then compare the results derived from the OSS, SB, NDO, PPDP, D3C, and PPDP+D3C methods. Finally, we present conclusions in Section 5.

## 2. Related techniques

In this section, we review the literature on the evaluation criteria for classification performance, the box-and-whisker plot method, and the MTD method.

### 2.1 Evaluation criteria

By convention, the minority class data is the positive class label, and the majority class data is the negative class label. For imbalanced class distributions, the accuracy rate for the minority class is frequently close to zero, which means that evaluations of learning results are not appropriate for use with minority class data. Consequently, the accuracy rate measure is not used to consider the classification performance in this work; instead, other criteria are described in this section. Table 1 shows a confusion matrix, which is used in this work to construct the relevant criteria for a two-class classification problem.

**Table 1:** Confusion matrix.

		Predicted class	
		Positive	Negative
Actual class	Positive	*TP*	*FN*
	Negative	*FP*	*TN*

The items in the confusion matrix are as follows: *TP* is the number of true positive examples; *FN* is the number of false negative examples; *FP* is the number of false positive examples; and *TN* is the number of true negative examples. The three criteria used in this study are defined as follows:

- Accuracy (ACC): $\text{ACC}=\frac{TP+TN}{TP+TN+FP+FN}$;
- Geometric mean (G-mean): $\text{G-mean}=\sqrt{\text{TPR}\times \text{TNR}}$, where $\text{TPR}=\frac{TP}{TP+FN}$ and $\text{TNR}=\frac{TN}{TN+FP}$;
- F-measure (F1): $\text{F}1=2\times \frac{\text{R}\times \text{P}}{\text{R}+\text{P}}$, where $\text{P}=\frac{TP}{TP+FP}$ and $\text{R}=\frac{TP}{TP+FN}$

### 2.2 Review of the box-and-whisker plot

The box-and-whisker technique was first proposed by Tukey [35] to show the distribution of data, examine its symmetry, and indicate outliers. Box-and-whisker plots are used to exclude outliers, where the box’s lower boundary is the lower quartile (*Q1*) of the data and the upper boundary is the upper quartile (*Q3*). The length of the box is the interquartile range (IQR), which is calculated by

IQR=Q3−Q1, (1)

where *Q3* and *Q1* are the 75th and 25th percentiles of the samples, respectively. In addition, *Q2* is the median of the data set. There are two inner fences in a box plot: the lower inner fence (*LIF*) and upper inner fence (*UIF*). When data are outside the [*LIF*,*UIF*], they are considered suspected outliers. The calculations for this region are as follows:

LIF=Q1−1.5×IQR, (2)

UIF=Q3+1.5×IQR. (3)

### 2.3 The MTD method

Li et al. [23] proposed the MTD method to construct the distribution of manufacturing data. The MTD method which combines mega diffusion and data trend estimation is used to generate virtual samples to provide a strategy for the knowledge of small data set learning and obtain a higher degree of classification accuracy.

**Fig 1:**
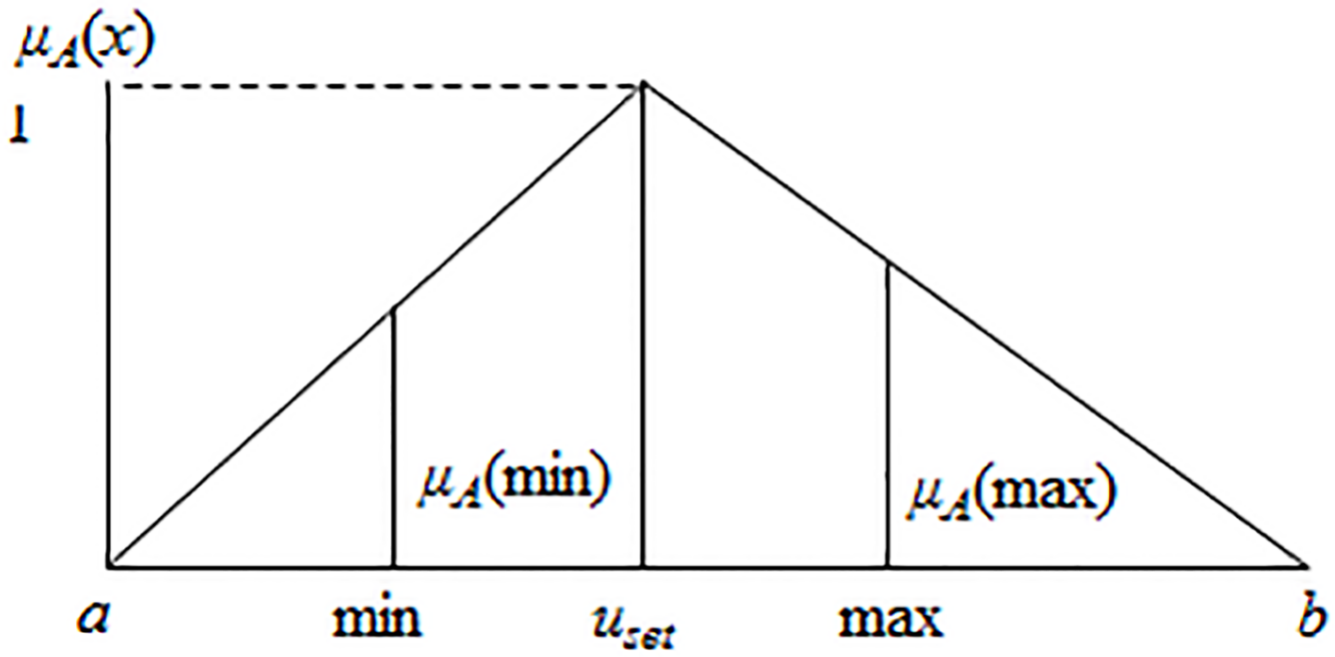
Data trend estimation.

As shown in Fig 1, a triangular membership function *μ*_*A*_(*x*) is constructed from the MTD method to calculate the domain range of observed/collected data *x*, which is the interval [*a*,*b*], described mathematically as:

$$a=u_{set}-S_{L}\times \sqrt{-2\times s_{x}^{2}/N_{L}\times \ln(10^{-20})},\;1<N_{L}<\infty ,\;\mathrm{and}$$ (4)

$$b=u_{set}+S_{U}\times \sqrt{-2\times s_{x}^{2}/N_{U}\times \ln(10^{-20})},\;1<N_{U}<\infty ,$$ (5)

where $S_{x}^{2}=\sum_{i=1}^{N}{\left(x_{i}-\bar{x}\right)}^{2}/\left(N-1\right)$ is the variance of observed data *x*_*i*_, *i* = 1,2,…,*N*, *N* is the sample size. *S*_*L*_ = *N*_*L*_/(*N*_*L*_ + *N*_*U*_) is the left of the skewness degree of $\sqrt{-2\times s_{x}^{2}/N_{L}\times \ln(10^{-20})}$ and *S*_*U*_ = *N*_*U*_/(*N*_*L*_ + *N*_*U*_) is the right of the skewness degree of $\sqrt{-2\times s_{x}^{2}/N_{U}\times \ln(10^{-20})}$. *N*_*L*_ and *N*_*U*_ indicate the number of data less than and greater than *u*_*set*_ that are equal to (min + max)/2, respectively, and “min” and “max” are the actual minimum and maximum values in the observed/collected data set. From Eqs (4) and (5), we can calculate the lower bound *a* and the upper bound *b*. That is, the values of *a* and *b* are the estimated domain range of observed/collected data set. Note that *a* = min/5 when *N*_*L*_ = 0 and *b* = max × 5 when *N*_*U*_ = 0. In addition, the related parameter settings are: *μ*_*A*_(*u*_*set*_) = 1, *μ*_*A*_(*a*) = *μ*_*A*_(*b*) = 0, *μ*_*A*_(min) = 1/*N*_*U*_ and *μ*_*A*_(max) = 1/*N*_*L*_.

## 3. The model structure

This section describes the proposed procedure to deal with imbalanced data set classification problems. It describes the undersampling process and explains the oversampling technique to find the shape of the data distribution with limited samples to generate synthetic samples for learning the skewed class distribution.

### 3.1 The proposed procedure

**Fig 2:**
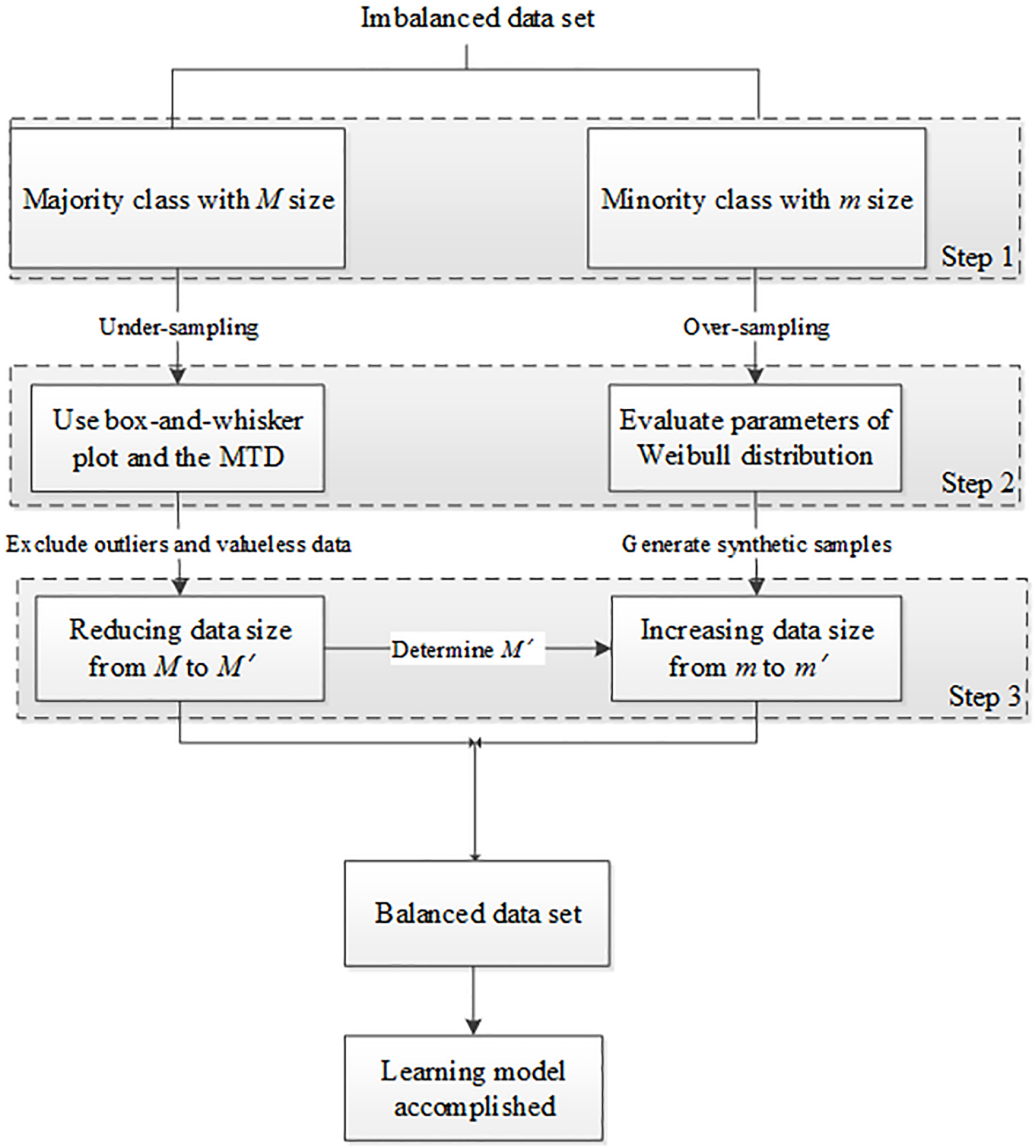
The proposed procedure for learning imbalanced data sets.

Fig 2 shows the detailed procedure of the proposed method, which contains three main steps.

In Step 1, the imbalanced data set is separated into two sets by class, where the majority class has *M* data and the minority class has *m* data. In Step 2, based on the undersampling strategy, we utilize the box-and-whisker plot to determine whether data are outliers in each feature. Then, we delete the outliers in the majority class. The MTD method is then applied to draw representative observations from the majority class. Regarding the oversampling strategy, because the number of samples in the minority class is small and may follow an arbitrary probability distribution, we consider the two-parameter Weibull distribution recommended by Little [36] to fit the data in the minority class and form various shapes of density functions, including skewed and mound-shaped curves, thus achieving greater flexibility. Therefore, by assuming that the minority class data are distributed into a two-parameter Weibull density function, we propose a method to evaluate the two parameters of the Weibull distribution and generate synthetic samples from that estimated distribution. In Step 3, given that these valuable parameters have been found and the data size in the majority class has been reduced from *M* to *M*', the size of synthetic data becomes *M*'−*m*, and we can then form the learning model by inputting the new balanced data set.

### 3.2 The undersampling method

The following method is proposed to rebuild the model of the data in the majority class. First, we employ the box-and-whisker plot to detect outliers and eliminate them from the majority data. Second, we use the remaining data to compute the range of the data, that is, the interval [*a*,*b*], as explained in Section 2.3. As shown in Fig 1, the triangular membership function *μ*_*A*_(*x*) is formed based on the interval [*a*,*b*], as follows:

$$\mu_{A}(x)=\{\begin{array}{c} \frac{x-a}{u_{set}-a},\;a\leq x\leq u_{set}\\ \frac{b-x}{b-u_{set}},\;u_{set}\leq x\leq b\\ 0,\;\text{otherwise} \end{array},$$ (6)

where *X* is assumed to be a universal set, and *x* is an element in *X*. The *A* set is a fuzzy set of *X*, and the value of *μ*_*A*_(*x*) is the membership function with regard to each *x* in [0,1].

Here, we apply theα-cut to draw the valuable data from the corresponding *μ*_*A*_(*x*) in *X*, where theα-cut of *A* is a crisp set that contains the total number of *x* in *X* that have values of *μ*_*A*_(*x*) greater than or equal toα-cut, denoted as follows:

$$A_{\alpha }=\left\{x\in X|\mu_{A}(x)\geq \alpha \text{-cut}\right\},\;\alpha \text{-cut}\in \left[0,1\right],$$

where *A*_*α*_ can be derived from Eq (6) as

$$A_{\alpha }=\left[(u_{set}-a)\times \alpha \text{-cut}+a,b-(b-u_{set})\times \alpha \text{-cut}\right].$$ (7)

We then use the data set in which all the data belong to *A*_*α*_ as a learning model for the majority class. In the majority class, when setting the value ofα-cut, we can implement this undersampling process to find the representative majority data.

### 3.3 The oversampling method

In this section, we first describe some basic properties of a two-parameter Weibull distribution, and then present the proposed method for oversampling in detail.

#### 3.3.1 Preparation for a two-parameter Weibull distribution

Given a data set *x* = {*x*_*i*_}, *i* = 1,2,⋯,*N* that can be denoted by a two-parameter Weibull distribution, the probability density function and cumulative distribution function of the Weibull distribution are respectively expressed as follows:

$$f(x,\lambda ,\beta )=\frac{\beta }{\lambda }{\left(\frac{x}{\lambda }\right)}^{\beta -1}\exp\left\{-{\left(\frac{x}{\lambda }\right)}^{\beta }\right\},x\geq 0\;,\lambda >0,\;\beta >0,\;\mathrm{and}$$ (8)

$$F(x,\lambda ,\beta )=1-\exp\left\{-{\left(\frac{x}{\lambda }\right)}^{\beta }\right\},\;x\geq 0,\;\lambda >0,\;\beta >0,$$ (9)

where *λ* is the scale parameter and *β* is the shape parameter.

With regard to the shape parameter, Nelson et al. [37] demonstrated that the Weibull distribution has some special expressions. For example, when the value of *β* is one or two, the Weibull distributions are identical to the Exponential and Rayleigh distributions, respectively, and the shape of the Weibull density function is close to a normal distribution when the value of *β* is within [3,4]. The least square estimation (LSE) is widely utilized by researchers to estimate the *β* and *λ* of Eq (8). The sum of squares error (SSE) can be derived from Eq (9) as

$$\text{SSE}=\sum_{i=1}^{N}{\left[\ln\left[-\ln(1-{\hat{F}}_{i}(x))\right]-\beta \ln x_{\left(i\right)}+\beta \ln\lambda \right]}^{2}$$ (10)

where *x*_(*i*)_ is the observed data, *i* = 1,⋯,*N*, *N* is the sample size, and the Bernard’s median rank estimator is ${\hat{F}}_{i}(x)=\left(i-0.3\right)/\left(N+0.4\right)$. This study executes the shape-first method to fit the optimal value of *β*. Then the different values of $\hat{\beta }$ are used to estimate *λ* based on the minimized SSE, as Eq (10).

#### 3.3.2 The estimation of the two parameters

The proposed method utilizes the Gini statistic [38] in counterintuitive hypothesis testing to find the best-fitting shape parameter *β* of the Weibull distribution. With a given level of significance *α* and a data size of *N*, the proposed testing procedure is constructed as follows:

Step 1 The null hypothesis is set to
  $$H_{0}:\beta =\beta_{0}.$$
Step 2 The alternative hypothesis is set to
  $$H_{1}:\beta \neq \beta_{0}.$$
Step 3 The testing statistic uses the Gini statistic as shown below:
  $$G_{N}=\sum_{i=1}^{N-1}i\times W_{i+1}/(N-1)\sum_{i=1}^{N}W_{i}$$ (11)
  where $W_{i}=(N-i+1)\times (x_{\left(i\right)}^{\beta }-x_{\left(i-1\right)}^{\beta })\;,\;i=1,2,\cdots ,N$, and *x*_(0)_ ≡ 0.
Step 4 **I**. The rejection region for a sample size of *N* between 3 and 20 is set to
  $$\{G_{N}>\xi_{1-\alpha /2}\}\;\mathrm{and}\;\{G_{N}<\xi_{\alpha /2}\},$$
  where the critical value *ξ*_*α*/2_ is the 100(*α*/2) percentile of the *G*_*N*_ statistic. Moreover, the *p*-value = *P*{|*G*_*N*_| > |*g*_*N*_||*β* = *β*_0_}, where *g*_*N*_ indicates the estimated value of *G*_*N*_, as follows:
  $$P(G_{N}\leq x)=x^{N-1}{\left\{\prod_{i=1}^{N-1}c_{i}\right\}}^{-1}-\sum_{j=m+1}^{N-1}{\left(x-c_{j}\right)}^{N-1}\cdot {\left\{c_{j}\prod_{k\neq j}^{N-1}\left(c_{k}-c_{j}\right)\right\}}^{-1}$$ (12)
  where *c*_*j*_ = (*N*−*j*)/(*N*−1), and *m* is the largest index, such that *x* ≤ *c*_*m*_. Note that the corresponding two-tailed percentiles *ξ*_*α*/2_ of the Gini statistic *G*_*N*_ are described in Gail and Gastwirth [38].
  **II**. The rejection region for a sample size of *N* that is greater than 20 is set to
  $$\left\{G_{N}>Z_{1-\alpha /2}\}\;\mathrm{and}\;\{G_{N}<Z_{\alpha /2}\right\},$$
  where *g*_*N*_ is the observed value of [12(*N*−1)]^1/2^ (*G*_*N*_−0.5) which follows an approximately standard normal distribution (normal(0,1)) expressed as shown below:
  $$P\{\left|Z\right|>\left|{\left[12(N-1)\right]}^{1/2}(g_{N}-0.5)\right||\beta =\beta_{0}\}$$ (13)
Step 5 The decision rule of the statistical test is designed as follows:
  When *β* = *β*_0_, the *p*-value has a maximal value, which means that there is strong evidence that the null hypothesis, *H*_0_ should be accepted. The best-fitting shape parameter *β* can be found based on this testing procedure. After *β* is estimated, we can compute the scale parameter *λ* using the following equation:
  $$\lambda =\exp\left\{-\frac{1}{\beta }\times \frac{1}{N}[\sum_{i=1}^{N}\left(\ln\{-\ln[1-{\hat{F}}_{i}(x)]\right\}-\beta \ln x_{i})]\right\},$$ (14)
  where Bernard’s median rank estimator is ${\hat{F}}_{i}(x)=\left(i-0.3\right)/\left(N+0.4\right)$, *i* = 1,⋯,*N*.

#### 3.3.3 Synthetic sample generation

As mentioned above, the minority class data are assumed to fit a two-parameter Weibull distribution. For a given data set, we employ the inversion method to derive the Weibull variate, which is the approach used here to create synthetic samples. In the inversion method, a random variable *X* is distributed in a Weibull distribution containing both a scale parameter *λ* and a shape parameter *β* (i.e., *X* ∼ Weibull(*λ*,*β*)). Given that *F*(*x*,*λ*,*β*) is the CDF of the data shown in Eq (9), it can be used to derive the formula of the Weibull variate as follows:

$$x=\lambda {\left\{-\ln[1-F(x,\lambda ,\beta )]\right\}}^{1/\beta },$$ (15)

where *x* ≥ 0, *λ* ≥ 0, *β* > 0. Subsequently, in the generation of the synthetic samples ${\hat{x}}_{1},\;{\hat{x}}_{2},\ldots {\hat{x}}_{N'}$, Eq (16) is modified to

$${\hat{x}}_{i}=\hat{\lambda }{\left\{-\ln[1-{\hat{F}}_{i}(x)]\right\}}^{1/\hat{\beta }}$$ (16)

where the Bernard’s median rank estimator ${\hat{F}}_{i}(x)=\left(i-0.3\right)/\left(N'+0.4\right)$, a desired number of *N*', and the two estimators $\hat{\lambda }$ and $\hat{\beta }$ are calculated by the proposed approach.

### 3.4 The detailed procedure

Assume that a training data set has *N* samples with *P* mutually independent features denoted as *T* = {(*X*_1_,*y*_1_),(*X*_2_,*y*_2_),…,(*X*_*N*_,*y*_*N*_)}, and the two-class data set where each sample *X*_*i*_, *i* = 1,…,*N* has *P* features (means *X*_*i*_ = (*x*_*i*1_, *x*_*i*2_,…*x*_*iP*_)), and *y*_*i*_ ∈ {+,−} is the target value of *X*_*i*_. Note that the class label of the minority class is positive (+), and the negative (−) label is for the majority data set. To explain the proposed procedure in detail, we provide the following steps:

Step 1 Separate the data set *T* into minority and majority data by the corresponding target value, denoted as $T=\{{\vec{t}}_{+},{\vec{t}}_{-}\}$, where ${\vec{t}}_{j}=\{I(y_{i}=j)(X_{1},j),\;I(y_{i}=j)(X_{2},j),\ldots ,\;I(y_{i}=j)(X_{N},j)\}$, *j* = {+,−} and *I*(⋅) is an indicator function that is selected if the condition in *I* holds and excluded otherwise.
Step 2 Use the box-and-whisker plot and the MTD method as the undersampling methods to exclude outliers and select the valuable data to reduce the data size of the majority class ${\vec{t}}_{-}$ from *M* to *M*'. Note that the number of items in the majority class becomes *M*', which can be calculated as *M*−*S*_*box*_−*S*_*mtd*_, where *S*_*box*_ and *S*_*mtd*_ are the quantity of outliers and valueless samples, respectively. *S*_*box*_ is the sample quantity that lies outside of [*LIF*,*UIF*], they are considered suspected outliers. *LIF* and *UIF* are shown as Eqs (2) and (3). *S*_*mtd*_ is the sample quantity that exceeds the value of *A*_*α*_, *A*_*α*_ = [(*u*_*set*_−*a*) × *α*-cut + *a*,*b*−(*b*−*u*_*set*_) × *α*-cut], given anα-cut. That means the data which exceeds the range of *A*_*α*_ will be removed.
Step 3 Utilize the oversampling method to increase the data size in the minority class ${\vec{t}}_{+}$ from *m* to *m*', where the number of synthetic samples in ${\vec{t}}_{+}$ is *M*'−*m*.
Step 4 The reduced ${\vec{t}}_{-}$ and extended ${\vec{t}}_{+}$ sets are merged into a new training data set to establish a learning model.

**Fig 3:**
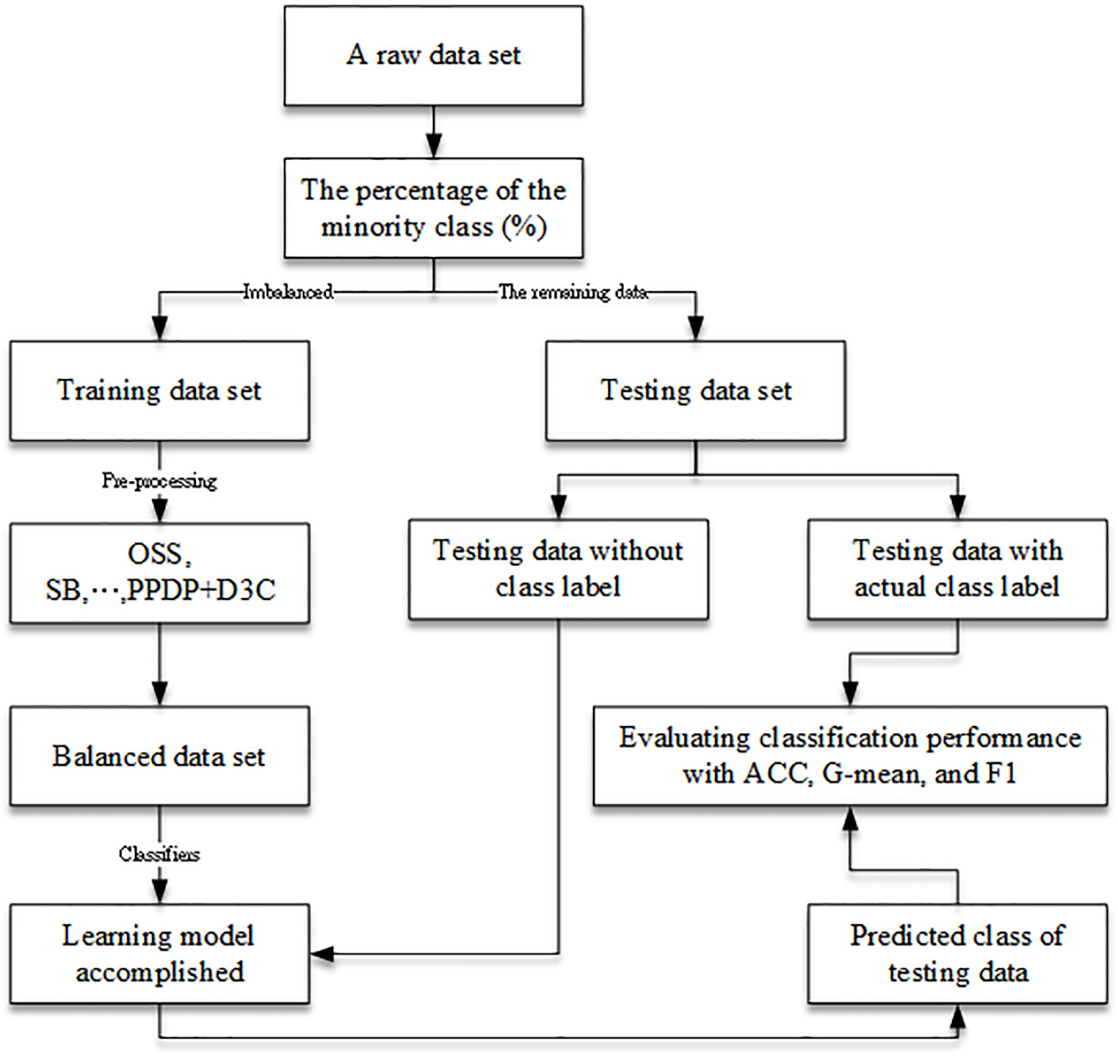
The testing procedure for imbalanced data sets.

For every data set, we can implement the above steps to balance the raw data set from (*M* + *m*) × *P* into (*M*' + *m*') × *P* dimensions. Besides, the remainder of the raw data set functioned as the testing data set. As for the testing procedure, this study will use the testing data and iterate the experiment 50 times concerning all of the scenarios given anα-cut to compare the result with that of the OSS, SB, NDO, PPDP, D3C methods. The testing procedure is shown as Fig 3.

## 4. Experiments

To demonstrate the classification performance of the PPDP+D3C method, we used four real data sets and compared the result with that of the OSS, SB, NDO, PPDP, D3C methods. Furthermore, paired t-tests were used in the comparison among them to examine the significance of the results with various sets of imbalanced data.

### 4.1 Four real data sets and classifier selection

In this section, we employ four real data sets (WDBC (available at: https://archive.ics.uci.edu/ml/datasets/Breast+Cancer+Wisconsin+%28Diagnostic%29), PD (available at: https://archive.ics.uci.edu/ml/datasets/parkinsons), VC (available at: http://archive.ics.uci.edu/ml/datasets/vertebral+column), and HS (available at: https://archive.ics.uci.edu/ml/datasets/Haberman's+Survival), downloaded from the UCI Machine Learning Repository database [39]) to demonstrate the performance of the PPDP+D3C with regard to the imbalanced two-class classification problems. The details of these four data sets are summarized in Table 2, where “*r*” indicates the percentage of minority classes in the samples.

**Table 2:** Data set description.

Data Set	No. Instances	No. Features	Feature Characteristics	*r*
WDBC	569	30	Numeric	37.26
PD	195	22	Numeric	24.62
VC	310	6	Numeric	32.22
HS	306	3	Numeric	26.47

This study applied four different classifiers including NB, KNN, and two types of SVM to the raw data of these four data sets. In KNN, the parameter of *k* was set to 3. The kernel functions in the two SVMs were linear and polynomial (notated as SVM-linear and SVM-poly, respectively); the cost parameter was set to 1 and the degree in kernel function set to 2 in the linear and polynomial kernel functions. The algorithms of the NB and KNN classifiers were implemented in Matlab, using the Statistics Toolbox. The SVM-linear and SVM-poly classifiers use LIBSVM [40] as the analysis tool. We selected the best classifier among the four classifiers in the imbalanced scenario (*r* = 5), using G-mean and F1 as the criteria for assessing classification performance with an imbalanced data set. Using the four raw data sets, we ran the experiment 50 times, set the training data size (*N*) as 60. The percentage of the minority classes was 5%, and the results, including ACC, G-mean, and F1, are shown in Table 3. The results show that the SVM-poly has a greater G-mean and F1 than NB, 3-NN, and SVM-linear. The bold values indicate that the SVM-poly achieved the best classification performance on both the WDBC, PD, VC, and HS data sets; it has the best G-mean and F1 scores.

**Table 3:** The results of four classifiers for the WDBC, PD, VC, and HS data set.

Data set	WDBC			
classifiers	NB	3-NN	SVM-linear	SVM-poly
ACC	61.39	59.63	**62.20**	61.12
G-mean	53.68	47.88	53.29	**66.19**
F1	49.71	44.14	49.34	**64.85**
Data set	PD			
classifiers	NB	3-NN	SVM-linear	SVM-poly
ACC	66.31	**67.73**	53.91	55.41
G-mean	51.71	10.13	37.30	**40.70**
F1	42.72	6.73	27.52	**31.80**
Data set	VC			
classifiers	NB	3-NN	SVM-linear	SVM-poly
ACC	**63.77**	62.25	49.15	48.56
G-mean	30.55	15.98	44.61	**46.66**
F1	20.56	7.32	37.21	**40.83**
Data set	HS			
classifiers	NB	3-NN	SVM-linear	SVM-poly
ACC	**68.88**	68.32	43.86	41.46
G-mean	18.57	7.62	28.75	**32.46**
F1	10.05	2.71	14.97	**19.92**

### 4.2 The suggested value ofα-cut

In the majority class, the value ofα-cut is important because it creates a region that controls the amount of representative data. To find an appropriate parameter setting forα-cut, we examined the classification performances of variousα-cut settings for both the WDBC, PD, VC, and HS data sets. According to the classifier selection results in Section 4.1, we utilized the SVM-poly classifier to analyze data based on the same parameter settings. Considering the imbalanced data set at (*r*,*N*) = (5,60) and performing the experiment 50 times, the results of the classifier's performance on ACC, G-mean, and F1 for different values ofα-cut are given in Table 4. As shown in Table 4, we can achieve better classification performance when the values of α-cut are 0.4 or 0.5. In our opinion, with a smallerα-cut, the data in the created region do not effectively represent the majority class, and the nature of the minority class gradually becomes fuzzy because of the corresponding increase in the number of synthetic samples *M*'−*m*. For other, higherα-cut values, the learning model may experience overfitting because the total amount of data (*M*' + *m*') becomes smaller. For this reason, we suggest that the value of α-cut should be set to 0.5.

**Table 4:** The results for differentα-cut values.

Data set	α-cut	0.1	0.2	0.3	0.4	0.5	0.6	0.7	0.8	0.9
WDBC	ACC	71.57	72.41	73.69	72.28	**75.24**	71.28	73.21	74.06	74.47
	G-mean	64.73	66.51	68.77	65.85	**71.28**	64.16	66.85	69.36	69.65
	F1	58.82	61.05	64.01	60.33	**67.11**	58.10	61.51	64.57	64.91
	*S*_*box*_	13	13	13	13	13	14	13	13	13
	*S*_*mtd*_	13	13	13	13	13	14	15	17	25
	*M’-m*	41	41	41	41	41	40	39	37	29
	*M’+m’*	88	88	88	88	88	86	84	80	64
Data set	α-cut	0.1	0.2	0.3	0.4	0.5	0.6	0.7	0.8	0.9
PD	ACC	67.25	67.61	64.84	67.21	**68.48**	64.53	67.62	67.70	67.64
	G-mean	60.03	61.08	58.60	59.19	**62.23**	57.46	60.81	59.72	60.35
	F1	50.27	51.54	48.00	49.21	**52.73**	46.60	51.11	50.03	50.61
	*S*_*box*_	10	10	10	10	11	11	10	10	10
	*S*_*mtd*_	10	10	11	10	12	12	14	19	29
	*M’-m*	44	44	43	44	42	42	40	35	25
	*M’+m’*	94	94	92	94	90	90	86	76	56
Data set	α-cut	0.1	0.2	0.3	0.4	0.5	0.6	0.7	0.8	0.9
VC	ACC	58.66	59.50	59.38	58.61	**59.85**	56.70	57.82	56.28	53.90
	G-mean	56.47	54.88	56.72	55.44	**57.82**	55.62	57.76	57.42	54.84
	F1	47.91	45.76	48.36	46.80	**49.79**	47.49	50.38	50.44	48.51
	*S*_*box*_	3	3	3	3	3	3	3	3	3
	*S*_*mtd*_	8	9	12	14	18	23	29	35	46
	*M’-m*	46	45	42	40	36	31	26	19	8
	*M’+m’*	98	96	90	86	78	68	58	44	22
Data set	α-cut	0.1	0.2	0.3	0.4	0.5	0.6	0.7	0.8	0.9
HS	ACC	57.51	57.11	55.30	**58.23**	57.67	57.84	55.49	54.63	52.35
	G-mean	51.39	51.11	49.46	**53.32**	51.71	51.87	50.64	50.18	48.04
	F1	38.29	38.18	36.54	**40.70**	38.92	39.08	37.93	37.37	36.66
	*S*_*box*_	6	7	6	6	6	6	6	6	6
	*S*_*mtd*_	16	18	22	24	27	31	37	40	50
	*M’-m*	38	36	32	30	27	23	17	14	4
	*M’+m’*	82	78	70	66	60	52	40	34	14

### 4.3 Experiment design

To create imbalanced scenarios, this experiment drew samples from a raw data set according to the percentage of the minority class, which was variously set to 5%, 10%, 15%, and 20% (*r* = {5,10,15,20}). The training data size, *N*, was set to 60, 80, 100 and 150 (a total of 16 scenarios). For example, when *r* = 5 and *N* = 60, there are four scenarios (*M*,*m*) = {(57,3),(76,4),(95,5),(142,8)}. The remainder of the raw data set functioned as the testing data set. Note that the minority class size must be at least three due to the limitation with regard to sample size described in Step 4 in Section 3.3.2. To comply with this restriction, any value of *r*(%)×*N* less than three was changed to three. Using the four data sets (WDBC, PD, VC and HS), we iterated this experiment 50 times (16 scenarios at one time) at α-cut = 0.5. The results in Tables 5, 6, 7 and 8 are the averages of the values of ACC, G-mean and F1 for the imbalanced data sets taken from the WDBC, PD, VC and HS data sets. We used the paired t-test to examine whether the PPDP+D3C achieved statistically significant superiority compared with those methods such as OSS, SB, NDO, PPDP, and D3C based on the ACC, G-mean and F1 measures. The statistical test results are listed in Tables 5, 6, 7 and 8. In these tables, the bold values indicate the highest values among the six methods, the values in the parentheses represent the P-value of paired t-test for PPDP+D3C and other mentioned methods. It shows PPDP+D3C had strong statistical significance (P-value < 0.05) with regard to its classification performance.

**Table 5:** The results of the six methods on WDBC dataset.

	*r* = 5	*N*							
	Method	60		80		100		150	
ACC	OSS	74.98	(0.00)	74.58	(0.00)	73.23	(0.00)	71.74	(0.00)
	SB	69.83	(0.00)	70.15	(0.00)	68.77	(0.00)	67.17	(0.00)
	NDO	74.01	(0.00)	74.87	(0.00)	73.19	(0.00)	71.21	(0.00)
	PPDP	73.51	(0.00)	74.37	(0.00)	72.38	(0.00)	71.42	(0.00)
	D3C	88.28	(0.00)	89.20	(0.03)	89.54	(0.09)	91.03	(0.03)
	PPDP+D3C	**89.83**	-	**89.95**	-	**90.30**	-	**91.85**	-
G-mean	OSS	68.01	(0.00)	68.89	(0.00)	68.33	(0.00)	69.40	(0.00)
	SB	59.53	(0.00)	62.13	(0.00)	61.27	(0.00)	63.19	(0.00)
	NDO	68.07	(0.00)	70.37	(0.00)	68.76	(0.00)	69.08	(0.00)
	PPDP	67.91	(0.00)	70.74	(0.00)	68.97	(0.00)	70.67	(0.00)
	D3C	85.85	(0.02)	87.37	(0.06)	88.29	(0.13)	90.91	(0.05)
	PPDP+D3C	**87.98**	-	**88.28**	-	**89.14**	-	**91.76**	-
F1	OSS	63.44	(0.00)	64.70	(0.00)	64.10	(0.00)	65.56	(0.00)
	SB	52.70	(0.00)	56.04	(0.00)	55.07	(0.00)	57.77	(0.00)
	NDO	63.12	(0.00)	66.29	(0.00)	64.42	(0.00)	65.18	(0.00)
	PPDP	62.66	(0.00)	66.50	(0.00)	64.46	(0.00)	66.98	(0.00)
	D3C	84.05	(0.01)	86.05	(0.04)	87.20	(0.13)	90.51	(0.05)
	PPDP+D3C	**86.63**	-	**87.14**	-	**88.17**	-	**91.41**	-
	*r* = 10	*N*							
	Method	60		80		100		150	
ACC	OSS	75.08	(0.00)	74.85	(0.00)	75.22	(0.00)	73.72	(0.00)
	SB	71.30	(0.00)	71.34	(0.00)	71.61	(0.00)	70.03	(0.00)
	NDO	74.82	(0.00)	74.99	(0.00)	75.14	(0.00)	72.81	(0.00)
	PPDP	73.96	(0.00)	75.02	(0.00)	74.63	(0.00)	74.36	(0.00)
	D3C	90.85	(0.03)	90.66	(0.08)	92.11	(0.58)	92.35	(0.00)
	PPDP+D3C	**91.77**	-	**91.33**	-	**92.31**	-	**93.62**	-
G-mean	OSS	69.00	(0.00)	70.00	(0.00)	71.54	(0.00)	71.92	(0.00)
	SB	63.50	(0.00)	64.92	(0.00)	66.66	(0.00)	67.40	(0.00)
	NDO	69.66	(0.00)	70.79	(0.00)	72.05	(0.00)	71.22	(0.00)
	PPDP	69.41	(0.00)	72.23	(0.00)	72.87	(0.00)	74.60	(0.00)
	D3C	89.57	(0.05)	89.48	(0.08)	91.55	(0.36)	92.26	(0.00)
	PPDP+D3C	**90.66**	-	**90.24**	-	**91.91**	-	**93.69**	-
F1	OSS	64.63	(0.00)	66.00	(0.00)	67.92	(0.00)	68.65	(0.00)
	SB	57.46	(0.00)	59.51	(0.00)	61.76	(0.00)	63.05	(0.00)
	NDO	65.12	(0.00)	66.82	(0.00)	68.47	(0.00)	67.81	(0.00)
	PPDP	64.55	(0.00)	68.25	(0.00)	69.15	(0.00)	71.74	(0.00)
	D3C	88.20	(0.04)	88.29	(0.08)	90.69	(0.41)	91.85	(0.00)
	PPDP+D3C	**89.48**	-	**89.15**	-	**91.05**	-	**93.36**	-
	*r* = 15	*N*							
	Method	60		80		100		150	
ACC	OSS	76.83	(0.00)	76.37	(0.00)	75.61	(0.00)	75.23	(0.00)
	SB	74.31	(0.00)	73.27	(0.00)	73.23	(0.00)	73.13	(0.00)
	NDO	77.10	(0.00)	76.42	(0.00)	75.04	(0.00)	75.22	(0.00)
	PPDP	75.99	(0.00)	75.62	(0.00)	75.41	(0.00)	76.45	(0.00)
	D3C	92.07	(0.03)	91.82	(0.13)	91.98	(0.02)	92.51	(0.00)
	PPDP+D3C	**92.78**	-	**92.45**	-	**92.81**	-	**93.67**	-
G-mean	OSS	71.89	(0.00)	72.43	(0.00)	72.12	(0.00)	73.67	(0.00)
	SB	68.32	(0.00)	67.77	(0.00)	69.10	(0.00)	71.04	(0.00)
	NDO	72.93	(0.00)	72.84	(0.00)	71.83	(0.00)	73.98	(0.00)
	PPDP	72.55	(0.00)	73.18	(0.00)	73.55	(0.00)	76.74	(0.00)
	D3C	90.82	(0.00)	91.07	(0.04)	91.03	(0.01)	92.24	(0.00)
	PPDP+D3C	**91.98**	-	**92.03**	-	**92.21**	-	**93.68**	-
F1	OSS	68.27	(0.00)	68.83	(0.00)	68.55	(0.00)	70.73	(0.00)
	SB	63.52	(0.00)	63.01	(0.00)	64.74	(0.00)	67.49	(0.00)
	NDO	69.22	(0.00)	69.29	(0.00)	68.11	(0.00)	71.09	(0.00)
	PPDP	68.40	(0.00)	69.36	(0.00)	69.90	(0.00)	74.18	(0.00)
	D3C	89.73	(0.01)	89.91	(0.06)	90.18	(0.01)	91.70	(0.00)
	PPDP+D3C	**90.88**	-	**90.88**	-	**91.37**	-	**93.22**	-
	*r* = 20	*N*							
	Method	60		80		100		150	
ACC	OSS	76.11	(0.00)	77.48	(0.00)	76.61	(0.00)	77.16	(0.00)
	SB	73.97	(0.00)	75.14	(0.00)	75.08	(0.00)	74.11	(0.00)
	NDO	76.25	(0.00)	77.70	(0.00)	76.97	(0.00)	75.64	(0.00)
	PPDP	75.76	(0.00)	77.13	(0.00)	77.17	(0.00)	76.17	(0.00)
	D3C	91.48	(0.00)	92.35	(0.00)	92.46	(0.07)	93.78	(0.03)
	PPDP+D3C	**92.78**	-	**93.05**	-	**92.99**	-	**94.33**	-
G-mean	OSS	71.33	(0.00)	74.26	(0.00)	73.61	(0.00)	75.98	(0.00)
	SB	68.60	(0.00)	70.90	(0.00)	71.75	(0.00)	71.92	(0.00)
	NDO	72.25	(0.00)	74.79	(0.00)	74.44	(0.00)	74.10	(0.00)
	PPDP	72.84	(0.00)	75.66	(0.00)	76.16	(0.00)	76.22	(0.00)
	D3C	90.22	(0.00)	91.45	(0.00)	91.65	(0.00)	93.57	(0.00)
	PPDP+D3C	**92.42**	-	**92.77**	-	**92.79**	-	**94.42**	-
F1	OSS	67.27	(0.00)	71.01	(0.00)	70.20	(0.00)	73.36	(0.00)
	SB	63.73	(0.00)	66.66	(0.00)	67.82	(0.00)	68.41	(0.00)
	NDO	68.25	(0.00)	71.45	(0.00)	71.15	(0.00)	71.08	(0.00)
	PPDP	68.67	(0.00)	72.23	(0.00)	72.94	(0.00)	73.39	(0.00)
	D3C	88.82	(0.00)	90.38	(0.00)	90.73	(0.01)	93.06	(0.01)
	PPDP+D3C	**91.07**	-	**91.59**	-	**91.71**	-	**93.87**	-

**Table 6:** The results of the six methods on PD dataset.

	*r* = 5	*N*							
	Method	60		80		100		150	
ACC	OSS	69.31	(0.00)	65.85	(0.00)	64.66	(0.00)	47.76	(0.00)
	SB	67.28	(0.00)	64.17	(0.00)	62.07	(0.00)	44.96	(0.00)
	NDO	67.47	(0.00)	66.74	(0.00)	65.17	(0.00)	53.40	(0.00)
	PPDP	67.86	(0.36)	65.92	(0.09)	63.93	(0.03)	51.53	(0.00)
	D3C	71.35	(0.00)	70.78	(0.01)	68.67	(0.01)	62.98	(0.03)
	PPDP+D3C	**73.73**	-	**72.71**	-	**70.82**	-	**66.76**	-
G-mean	OSS	55.09	(0.00)	54.39	(0.00)	58.89	(0.00)	62.99	(0.00)
	SB	50.31	(0.00)	50.62	(0.00)	54.79	(0.00)	59.97	(0.00)
	NDO	60.54	(0.00)	61.18	(0.00)	62.50	(0.00)	64.91	(0.00)
	PPDP	58.88	(0.00)	61.61	(0.00)	61.86	(0.00)	66.24	(0.00)
	D3C	62.84	(0.01)	65.02	(0.02)	65.54	(0.00)	69.75	(0.02)
	PPDP+D3C	**66.71**	-	**67.62**	-	**68.72**	-	**73.54**	-
F1	OSS	45.53	(0.00)	45.33	(0.00)	52.12	(0.00)	61.58	(0.00)
	SB	39.48	(0.00)	40.69	(0.00)	46.84	(0.00)	59.19	(0.00)
	NDO	50.83	(0.00)	53.39	(0.00)	56.97	(0.00)	67.40	(0.00)
	PPDP	49.04	(0.00)	53.70	(0.00)	56.05	(0.00)	66.58	(0.00)
	D3C	52.81	(0.00)	57.48	(0.02)	60.59	(0.00)	75.52	(0.03)
	PPDP+D3C	**57.52**	-	**60.56**	-	**64.36**	-	**78.80**	-
	*r* = 10	*N*							
	Method	60		80		100		150	
ACC	OSS	71.99	(0.05)	70.72	(0.00)	70.77	(0.32)	66.56	(0.46)
	SB	69.67	(0.00)	69.66	(0.00)	68.33	(0.00)	62.33	(0.00)
	NDO	69.68	(0.00)	70.93	(0.00)	68.47	(0.00)	63.22	(0.00)
	PPDP	68.44	(0.76)	70.57	(0.03)	67.44	(0.44)	62.47	(0.90)
	D3C	73.69	(0.95)	**74.19**	(0.30)	**73.33**	(0.01)	**69.82**	(0.03)
	PPDP+D3C	**73.73**	-	73.60	-	71.64	-	67.64	-
G-mean	OSS	62.29	(0.00)	66.01	(0.00)	67.92	(0.05)	72.66	(0.16)
	SB	56.79	(0.00)	62.42	(0.00)	64.04	(0.00)	69.42	(0.35)
	NDO	64.77	(0.00)	68.32	(0.01)	66.69	(0.00)	69.39	(0.37)
	PPDP	63.78	(0.02)	69.02	(0.77)	66.90	(0.33)	69.94	(0.37)
	D3C	66.25	(0.03)	70.41	(0.69)	**71.49**	(0.05)	**73.84**	(0.01)
	PPDP+D3C	**68.51**	-	**70.73**	-	69.92	-	70.63	-
F1	OSS	53.30	(0.03)	58.55	(0.01)	62.64	(0.45)	74.34	(0.57)
	SB	46.75	(0.00)	54.37	(0.00)	57.73	(0.00)	70.18	(0.00)
	NDO	55.18	(0.09)	61.19	(0.37)	61.02	(0.04)	71.43	(0.00)
	PPDP	53.72	(0.06)	61.75	(0.68)	61.16	(0.27)	71.68	(0.08)
	D3C	56.22	(0.05)	62.78	(0.64)	**66.35**	(0.07)	**77.70**	(0.08)
	PPDP+D3C	**58.56**	-	**63.23**	-	64.55	-	76.14	-
	*r* = 15	*N*							
	Method	60		80		100		150	
ACC	OSS	72.63	(0.71)	71.24	(0.08)	73.48	(0.30)	71.04	(0.27)
	SB	71.93	(0.32)	71.89	(0.27)	71.72	(0.49)	70.07	(0.66)
	NDO	71.36	(0.09)	72.86	(0.96)	71.91	(0.61)	70.02	(0.67)
	PPDP	69.81	(0.08)	69.68	(0.05)	70.22	(0.00)	67.76	(0.01)
	D3C	**75.39**	(0.00)	**75.72**	(0.00)	**74.88**	(0.00)	**71.91**	(0.04)
	PPDP+D3C	72.99	-	72.82	-	72.38	-	69.47	-
G-mean	OSS	67.38	(0.02)	66.92	(0.00)	71.74	(0.31)	73.79	(0.12)
	SB	63.63	(0.00)	65.82	(0.00)	67.71	(0.02)	73.463	(0.18)
	NDO	66.95	(0.00)	70.24	(0.29)	70.26	(0.83)	73.31	(0.19)
	PPDP	66.96	(0.33)	68.59	(0.58)	70.17	(0.09)	71.74	(0.07)
	D3C	**71.41**	(0.09)	**72.27**	(0.29)	**72.30**	(0.04)	**74.65**	(0.01)
	PPDP+D3C	70.04	-	71.17	-	70.49	-	71.60	-
F1	OSS	57.45	(0.91)	58.32	(0.08)	65.81	(0.02)	73.38	(0.28)
	SB	53.55	(0.01)	57.57	(0.01)	61.07	(0.42)	73.78	(0.42)
	NDO	56.56	(0.40)	62.36	(0.19)	63.93	(0.18)	74.03	(0.30)
	PPDP	56.14	(0.73)	59.74	(0.90)	63.47	(0.07)	73.10	(0.22)
	D3C	**60.71**	(0.04)	**63.63**	(0.17)	**65.56**	(0.03)	**74.96**	(0.33)
	PPDP+D3C	58.63	-	61.91	-	63.24	-	73.62	-
	*r* = 20	*N*							
	Method	60		80		100		150	
ACC	OSS	74.05	(0.62)	74.70	(0.18)	74.96	(0.14)	73.67	(0.08)
	SB	73.40	(0.83)	74.65	(0.10)	74.39	(0.32)	75.40	(0.00)
	NDO	72.30	(0.20)	74.20	(0.26)	74.28	(0.34)	74.96	(0.01)
	PPDP	70.51	(0.09)	71.92	(0.00)	72.28	(0.00)	73.22	(0.00)
	D3C	**76.19**	(0.00)	**77.63**	(0.00)	**78.51**	(0.00)	**75.64**	(0.00)
	PPDP+D3C	73.59	-	73.41	-	73.58	-	71.20	-
G-mean	OSS	70.97	(0.33)	71.78	(0.80)	73.88	(0.05)	75.35	(0.04)
	SB	67.11	(0.00)	70.04	(0.15)	72.14	(0.90)	**77.12**	(0.00)
	NDO	69.92	(0.06)	72.02	(0.51)	73.90	(0.02)	76.59	(0.00)
	PPDP	69.12	(0.40)	71.44	(0.04)	73.12	(0.00)	75.89	(0.00)
	D3C	**72.21**	(0.85)	**73.70**	(0.01)	**76.01**	(0.00)	76.52	(0.00)
	PPDP+D3C	72.05	-	71.47	-	72.03	-	72.23	-
F1	OSS	**60.67**	(0.12)	63.03	(0.03)	66.56	(0.00)	74.97	(0.01)
	SB	56.65	(0.16)	61.43	(0.12)	64.59	(0.02)	**76.80**	(0.00)
	NDO	58.89	(0.80)	63.03	(0.00)	66.23	(0.00)	76.51	(0.00)
	PPDP	57.57	(0.98)	61.76	(0.01)	64.96	(0.00)	76.15	(0.00)
	D3C	60.64	(0.34)	**64.27**	(0.00)	**68.10**	(0.00)	75.03	(0.00)
	PPDP+D3C	59.53	-	60.51	-	62.71	-	71.28	-

**Table 7:** The results of the six methods on VC dataset.

	*r* = 5	*N*							
	Method	60		80		100		150	
ACC	OSS	58.70	(0.00)	58.68	(0.00)	58.91	(0.00)	59.49	(0.00)
	SB	58.92	(0.00)	60.89	(0.00)	60.62	(0.00)	62.34	(0.00)
	NDO	62.76	(0.00)	63.30	(0.00)	64.56	(0.00)	66.84	(0.01)
	PPDP	59.17	(0.00)	58.97	(0.00)	60.59	(0.00)	60.04	(0.00)
	D3C	**67.84**	(0.65)	**67.66**	(0.37)	**67.42**	(0.85)	67.38	(0.15)
	PPDP+D3C	67.36	-	66.90	-	67.26	-	**68.73**	-
G-mean	OSS	52.71	(0.00)	55.49	(0.00)	57.71	(0.00)	61.88	(0.00)
	SB	50.60	(0.00)	56.36	(0.00)	58.48	(0.00)	64.84	(0.00)
	NDO	59.86	(0.00)	62.02	(0.00)	64.97	(0.05)	**69.06**	(0.54)
	PPDP	56.74	(0.00)	58.39	(0.00)	62.03	(0.00)	62.93	(0.00)
	D3C	62.26	(0.24)	63.36	(0.44)	64.46	(0.06)	67.95	(0.47)
	PPDP+D3C	**64.07**	-	**64.53**	-	**66.68**	-	68.61	-
F1	OSS	43.41	(0.00)	48.05	(0.00)	52.21	(0.00)	61.70	(0.00)
	SB	40.49	(0.00)	48.56	(0.00)	52.49	(0.00)	64.65	(0.00)
	NDO	51.99	(0.01)	55.91	(0.03)	61.16	(0.07)	70.62	(0.44)
	PPDP	47.96	(0.00)	51.71	(0.00)	58.20	(0.00)	64.98	(0.00)
	D3C	54.43	(0.24)	57.06	(0.30)	59.48	(0.00)	68.15	(0.00)
	PPDP+D3C	**56.54**	-	**59.00**	-	**64.12**	-	**72.24**	-
	*r* = 10	*N*							
	Method	60		80		100		150	
ACC	OSS	60.02	(0.00)	58.26	(0.00)	61.62	(0.00)	63.89	(0.00)
	SB	60.74	(0.00)	60.33	(0.00)	64.40	(0.00)	67.36	(0.00)
	NDO	63.68	(0.00)	65.34	(0.00)	67.30	(0.07)	68.93	(0.00)
	PPDP	60.21	(0.00)	59.71	(0.00)	62.10	(0.03)	63.46	(0.00)
	D3C	**68.75**	(0.48)	**69.64**	(0.45)	**71.58**	(0.00)	71.06	(0.97)
	PPDP+D3C	68.33	-	69.00	-	68.42	-	**71.09**	-
G-mean	OSS	58.13	(0.00)	57.56	(0.00)	62.70	(0.00)	65.72	(0.00)
	SB	56.91	(0.00)	58.92	(0.00)	65.04	(0.00)	69.29	(0.04)
	NDO	62.92	(0.00)	66.09	(0.01)	68.51	(0.77)	70.88	(0.95)
	PPDP	59.55	(0.00)	60.65	(0.00)	64.03	(0.00)	65.93	(0.00)
	D3C	63.92	(0.01)	64.19	(0.00)	**69.08**	(0.44)	**71.12**	(0.77)
	PPDP+D3C	**66.76**	-	**68.65**	-	68.30	-	70.92	-
F1	OSS	49.84	(0.00)	50.80	(0.00)	58.84	(0.00)	66.99	(0.00)
	SB	47.89	(0.00)	51.80	(0.00)	60.93	(0.01)	70.19	(0.00)
	NDO	55.64	(0.00)	60.81	(0.06)	**65.11**	(0.26)	71.77	(0.11)
	PPDP	51.57	(0.00)	54.40	(0.00)	60.34	(0.00)	68.06	(0.00)
	D3C	55.85	(0.01)	57.07	(0.00)	64.34	(0.56)	69.64	(0.00)
	PPDP+D3C	**59.91**	-	**63.85**	-	65.06	-	**73.79**	-
	*r* = 15	*N*							
	Method	60		80		100		150	
ACC	OSS	59.06	(0.00)	61.15	(0.00)	63.71	(0.00)	65.56	(0.00)
	SB	62.22	(0.00)	63.81	(0.00)	65.85	(0.00)	68.45	(0.00)
	NDO	64.61	(0.00)	67.20	(0.00)	68.68	(0.22)	69.23	(0.03)
	PPDP	60.26	(0.00)	62.42	(0.00)	62.49	(0.00)	63.59	(0.00)
	D3C	71.95	(0.00)	72.88	(0.00)	73.46	(0.00)	72.91	(0.00)
	PPDP+D3C	**68.63**	-	**69.72**	-	**69.32**	-	**70.04**	-
G-mean	OSS	59.15	(0.00)	62.03	(0.00)	65.11	(0.00)	67.25	(0.00)
	SB	60.95	(0.00)	63.50	(0.00)	66.87	(0.00)	70.23	(0.98)
	NDO	65.34	(0.00)	67.95	(0.00)	70.17	(0.82)	71.04	(0.03)
	PPDP	61.03	(0.00)	64.21	(0.00)	65.27	(0.00)	66.26	(0.00)
	D3C	66.31	(0.16)	69.38	(0.66)	71.13	(0.19)	72.46	(0.00)
	PPDP+D3C	**68.31**	-	**69.71**	-	**70.04**	-	**70.24**	-
F1	OSS	51.17	(0.00)	56.07	(0.00)	60.96	(0.00)	67.73	(0.00)
	SB	52.63	(0.00)	56.95	(0.00)	62.35	(0.00)	70.36	(0.86)
	NDO	58.20	(0.04)	62.32	(0.17)	66.16	(0.50)	71.34	(0.04)
	PPDP	53.16	(0.00)	58.10	(0.00)	61.76	(0.00)	67.28	(0.00)
	D3C	58.38	(0.06)	63.11	(0.19)	65.96	(0.42)	70.46	(0.22)
	PPDP+D3C	**61.36**	-	**64.34**	-	**66.77**	-	**71.46**	-
	*r* = 20	*N*							
	Method	60		80		100		150	
ACC	OSS	60.69	(0.00)	60.09	(0.00)	61.42	(0.00)	66.19	(0.00)
	SB	63.57	(0.00)	63.83	(0.00)	66.00	(0.00)	69.40	(0.01)
	NDO	65.23	(0.00)	66.46	(0.00)	67.76	(0.00)	70.69	(0.12)
	PPDP	60.93	(0.00)	61.52	(0.00)	62.80	(0.00)	65.20	(0.03)
	D3C	**73.38**	(0.00)	**73.87**	(0.00)	**74.93**	(0.00)	**74.45**	(0.00)
	PPDP+D3C	69.34	-	69.59	-	70.68	-	71.51	-
G-mean	OSS	62.23	(0.00)	62.24	(0.00)	63.57	(0.00)	68.06	(0.00)
	SB	63.52	(0.00)	64.62	(0.00)	67.54	(0.00)	71.25	(0.30)
	NDO	66.10	(0.00)	68.18	(0.00)	69.50	(0.00)	72.61	(0.27)
	PPDP	62.63	(0.00)	64.14	(0.00)	65.57	(0.00)	68.04	(0.00)
	D3C	68.62	(0.50)	69.93	(0.78)	**72.53**	(0.22)	**72.91**	(0.36)
	PPDP+D3C	**69.37**	-	**70.25**	-	71.60	-	72.01	-
F1	OSS	54.73	(0.00)	56.57	(0.00)	59.05	(0.00)	67.68	(0.00)
	SB	55.44	(0.00)	57.94	(0.00)	62.41	(0.00)	70.34	(0.57)
	NDO	58.62	(0.01)	62.32	(0.05)	64.68	(0.00)	71.56	(0.14)
	PPDP	54.72	(0.00)	57.94	(0.00)	60.76	(0.00)	67.45	(0.00)
	D3C	60.83	(0.31)	63.19	(0.24)	66.96	(0.65)	69.69	(0.08)
	PPDP+D3C	**62.18**	-	**64.73**	-	**67.36**	-	**71.75**	-

**Table 8:** The results of the six methods on HS dataset.

	*r* = 5	*N*							
	Method	60		80		100		150	
ACC	OSS	59.46	(0.53)	56.78	(0.06)	56.48	(0.03)	52.44	(0.00)
	SB	61.06	(0.54)	57.72	(0.35)	57.01	(0.04)	52.74	(0.01)
	NDO	60.87	(0.62)	57.18	(0.11)	58.30	(0.28)	55.14	(0.27)
	PPDP	56.25	(0.27)	54.05	(0.66)	54.90	(0.50)	50.35	(0.32)
	D3C	**66.07**	(0.00)	**63.62**	(0.00)	**64.72**	(0.00)	**58.86**	(0.03)
	PPDP+D3C	60.33	-	58.71	-	59.29	-	56.32	-
G-mean	OSS	44.36	(0.00)	45.82	(0.00)	47.27	(0.00)	49.22	(0.00)
	SB	42.20	(0.00)	43.56	(0.00)	46.20	(0.00)	49.24	(0.00)
	NDO	48.43	(0.01)	49.08	(0.24)	50.67	(0.00)	52.88	(0.25)
	PPDP	51.08	(0.98)	48.62	(0.67)	51.89	(0.34)	49.55	(0.03)
	D3C	49.50	(0.08)	45.83	(0.00)	48.08	(0.00)	46.46	(0.00)
	PPDP+D3C	**53.05**	-	**51.28**	-	**55.21**	-	**54.41**	-
F1	OSS	30.53	(0.00)	32.87	(0.01)	35.69	(0.00)	41.63	(0.00)
	SB	28.27	(0.00)	30.27	(0.00)	34.16	(0.00)	41.37	(0.00)
	NDO	35.42	(0.03)	37.08	(0.47)	39.90	(0.01)	46.32	(0.11)
	PPDP	38.48	(0.82)	36.60	(0.56)	41.64	(0.17)	43.22	(0.01)
	D3C	36.62	(0.05)	33.30	(0.00)	35.02	(0.00)	36.40	(0.00)
	PPDP+D3C	**40.90**	-	**41.69**	-	**45.98**	-	**49.93**	-
	*r* = 10	*N*							
	Method	60		80		100		150	
ACC	OSS	58.27	(0.08)	58.07	(0.00)	57.64	(0.00)	56.05	(0.00)
	SB	59.21	(0.29)	58.88	(0.00)	58.58	(0.00)	57.94	(0.00)
	NDO	60.10	(0.82)	60.74	(0.00)	60.76	(0.32)	58.79	(0.00)
	PPDP	54.66	(0.46)	58.72	(0.62)	55.58	(0.16)	54.92	(0.00)
	D3C	**67.91**	(0.00)	**68.07**	(0.00)	**67.81**	(0.00)	**63.04**	(0.09)
	PPDP+D3C	60.33	-	64.05	-	61.63	-	61.82	-
G-mean	OSS	46.66	(0.00)	50.16	(0.00)	52.19	(0.00)	53.26	(0.00)
	SB	44.88	(0.00)	48.45	(0.00)	52.36	(0.00)	55.46	(0.00)
	NDO	50.25	(0.00)	52.48	(0.00)	53.27	(0.00)	55.04	(0.00)
	PPDP	50.51	(0.13)	54.72	(0.02)	53.86	(0.00)	54.34	(0.00)
	D3C	48.38	(0.00)	47.88	(0.00)	49.70	(0.00)	49.68	(0.00)
	PPDP+D3C	**54.56**	-	**59.43**	-	**59.66**	-	**60.32**	-
F1	OSS	32.53	(0.00)	37.41	(0.00)	40.86	(0.00)	45.44	(0.00)
	SB	30.56	(0.00)	35.36	(0.00)	40.87	(0.00)	48.04	(0.00)
	NDO	36.77	(0.01)	40.27	(0.00)	42.28	(0.00)	47.57	(0.00)
	PPDP	37.05	(0.07)	42.58	(0.01)	43.17	(0.00)	47.23	(0.00)
	D3C	35.10	(0.00)	36.60	(0.00)	37.98	(0.00)	39.37	(0.00)
	PPDP+D3C	**41.76**	-	**48.32**	-	**50.55**	-	**55.24**	-
	*r* = 15	*N*							
	Method	60		80		100		150	
ACC	OSS	56.58	(0.00)	57.30	(0.00)	57.14	(0.00)	58.88	(0.00)
	SB	58.41	(0.00)	59.63	(0.00)	58.76	(0.00)	59.99	(0.00)
	NDO	61.19	(0.03)	62.78	(0.71)	62.49	(0.00)	61.50	(0.00)
	PPDP	55.55	(0.00)	54.75	(0.00)	56.96	(0.00)	55.58	(0.00)
	D3C	**70.77**	(0.00)	**69.88**	(0.00)	**69.91**	(0.00)	**65.47**	(0.00)
	PPDP+D3C	63.24	-	63.10	-	65.15	-	63.67	-
G-mean	OSS	50.50	(0.00)	50.18	(0.00)	51.19	(0.00)	56.49	(0.00)
	SB	49.46	(0.00)	50.15	(0.00)	50.96	(0.00)	57.01	(0.00)
	NDO	52.97	(0.00)	53.71	(0.00)	54.11	(0.00)	54.85	(0.00)
	PPDP	51.48	(0.00)	51.44	(0.00)	54.50	(0.00)	54.99	(0.00)
	D3C	49.46	(0.00)	45.30	(0.00)	49.54	(0.00)	49.69	(0.00)
	PPDP+D3C	**59.46**	-	**60.00**	-	**62.18**	-	**61.36**	-
F1	OSS	35.99	(0.00)	36.65	(0.00)	38.59	(0.00)	48.09	(0.00)
	SB	34.84	(0.00)	36.68	(0.00)	38.29	(0.00)	48.54	(0.00)
	NDO	39.07	(0.00)	41.20	(0.00)	42.29	(0.00)	45.49	(0.00)
	PPDP	37.32	(0.00)	38.16	(0.00)	42.59	(0.00)	46.62	(0.00)
	D3C	31.54	(0.00)	32.47	(0.00)	37.52	(0.00)	38.64	(0.00)
	PPDP+D3C	**46.63**	-	**48.66**	-	**51.56**	-	**54.09**	-
	*r* = 20	*N*							
	Method	60		80		100		150	
ACC	OSS	56.25	(0.00)	58.23	(0.00)	57.41	(0.00)	59.78	(0.00)
	SB	59.24	(0.00)	60.13	(0.00)	59.98	(0.00)	60.63	(0.00)
	NDO	61.76	(0.01)	63.73	(0.01)	62.62	(0.00)	62.32	(0.00)
	PPDP	55.72	(0.00)	58.63	(0.00)	57.33	(0.00)	57.32	(0.00)
	D3C	**71.11**	(0.00)	**71.09**	(0.00)	**70.59**	(0.00)	**67.53**	(0.00)
	PPDP+D3C	64.04	-	66.13	-	65.09	-	65.42	-
G-mean	OSS	50.56	(0.00)	52.48	(0.00)	54.39	(0.00)	57.93	(0.00)
	SB	50.65	(0.00)	51.28	(0.00)	55.97	(0.00)	57.28	(0.00)
	NDO	53.80	(0.00)	54.26	(0.00)	55.56	(0.00)	55.63	(0.00)
	PPDP	52.34	(0.00)	54.98	(0.00)	55.03	(0.00)	56.29	(0.00)
	D3C	48.40	(0.00)	45.64	(0.00)	50.34	(0.00)	48.24	(0.00)
	PPDP+D3C	**60.68**	-	**61.23**	-	**61.89**	-	**62.05**	-
F1	OSS	35.78	(0.00)	38.53	(0.00)	41.46	(0.00)	48.34	(0.00)
	SB	35.83	(0.00)	37.15	(0.00)	43.20	(0.00)	47.40	(0.00)
	NDO	39.72	(0.00)	41.23	(0.00)	43.02	(0.00)	45.39	(0.00)
	PPDP	37.79	(0.00)	41.44	(0.00)	42.18	(0.00)	46.45	(0.00)
	D3C	35.06	(0.00)	32.52	(0.00)	37.68	(0.00)	36.45	(0.00)
	PPDP+D3C	**47.19**	-	**48.68**	-	**49.97**	-	**53.02**	-

### 4.4 Experiment results

The experiment results are listed in Tables 5, 6, 7 and 8 for the 16 scenarios (*r* = {5,10,15,20} and *N* = {60,80,100,150}). For instance, with (*r*,*N*) = (5,60) in the WDBC data set, the PPDP+D3C achieved a better classification performance than the D3C method; the differences of the G-mean and F1 are 87.98–85.85 = 2.13(%) and 86.63–84.05 = 2.58(%) with a group of class sizes (*M*,*m*) = (57,3), respectively, and the P-values based on the G-mean and F1 are smaller than 0.05. From Tables 5, 6, 7 and 8, the comparative results of above-mentioned methods are as follows:

1. For a fixed *N* and *r*, the OSS, SB, NDO, PPDP methods have very close ACC values, and there are no statistically significant differences between the other five methods and PPDP+D3C.
2. For a fixed *N*, when *r* is increasing, the values of G-mean and F1 increase for all six methods, and the improvement in ACC is not significant.
3. For a fixed *N*, when *r* has become large, the PPDP+D3C consistently achieves better G-mean and F1 scores than the other five methods do, although this superiority is not statistically significant with regard to D3C in a few scenarios.
4. For a fixed *r*, when *N* is increasing, the PPDP+D3C consistently achieves higher G-mean and F1 scores than D3C does in the most scenarios.
5. For a small *r* and *N*, the results of the paired t-tests between PPDP+D3C and D3C are significant with regard to G-mean and F1 in the some scenarios.

As shown in Tables 5, 6, 7 and 8, we can see in the WDBC dataset the performance of PPDP+D3C in ACC, G-mean and F1 is better compared to the other methods. In other data sets, the D3C can achieve better performance concerning ACC, but less statistically significant differences. While the PPDP+D3C method achieves excellent performance concerning G-mean and F1 compared to the other methods and most of which have statistically significant differences. These results show that the PPDP+D3C method achieves higher classification performance on imbalanced data sets among the other five tested methods. In other words, the results show that the proposed method can effectively achieve better classification performance with small values of *r* and *N*. Thus, it is obvious that when a data set includes imbalanced classes, the classification performance can be significantly improved by using the PPDP+D3C method. In addition, for a fixed *N*, when *r* is increasing, the number of generated synthetic samples *M*'−*m* becomes smaller, as shown in Table 9.

**Table 9:** The number of *S*_*box*_, *S*_*mtd*_, *M*'−*m*, and *M*'+*m*' with *N* = 60.

Dataset	*N* = 60				Dataset	*N* = 60			
WDBC					PD				
*r*	5	10	15	20	*r*	5	10	15	20
*S*_*box*_	13	13	12	12	*S*_*box*_	10	10	9	9
*S*_*mtd*_	14	13	12	12	*S*_*mtd*_	12	11	10	10
*M*'−*m*	40	37	32	28	*M*'−*m*	42	39	34	30
*M*'+*m*'	86	84	80	76	*M*'+*m*'	90	88	84	80
Dataset	*N* = 60				Dataset	*N* = 60			
VC					HS				
*r*	5	10	15	20	*r*	5	10	15	20
*S*_*box*_	3	3	3	3	*S*_*box*_	6	6	6	6
*S*_*mtd*_	18	18	16	14	*S*_*mtd*_	27	26	24	23
*M*'−*m*	36	32	28	26	*M*'−*m*	27	24	20	17
*M*'+*m*'	78	74	72	72	*M*'+*m*'	60	58	56	54

### 4.5 Summary and discussion

Four data sets, the WDBC, PD, VC, and HS, were used in this research to show the performance of the PPDP+D3C method with regard to learning with imbalanced data sets. Based on the results of experiments, as shown in Tables 5, 6, 7 and 8, the findings can be summarized as follows. The merging of the PPDP+D3C achieves better classification performance than the other five methods, and this superiority is statistically significant. In a few scenarios, when the value of *r* is 15 or 20 with a larger *N*, the PPDP+D3C has better G-mean and F1 scores than those of the D3C method, although some comparisons of the results of the paired t-test between the PPDP+D3C and the D3C showed no significant differences with regard to the G-mean and F1 scores. This may be because the ratio of minority data to the overall samples is rather large, and the amount of data in the minority part is thus sufficient for learning to occur based on the minority class. For instance, in the VC data set, the P-value of the paired t-test for F1 is 0.08, which is greater than 0.05 at (*r*,*N*) = (20,150). In fact, the results in Tables 5, 6, 7 and 8 show that when the value of *r*(%)×*N* is smaller, the PPDP+D3C method improves significantly in classification performance with regard to the G-mean and F1 measures.

## 5. Conclusion

Imbalanced data classification problems are common in the field of data mining, often leading to low classification performances because the existing learning algorithms are more suitable for the majority class data. In this work, we combined undersampling and oversampling to balance the training data sets; the undersampling method uses the box-and-whisker plot and the MTD method to reduce the size of the majority class data, while the oversampling method extends the minority class data set by adding generated synthetic samples. Experiments were carried out based on four imbalanced data sets. In particular, imbalanced data of a certain disease may differ based on different region, era, and medical environment. It leads to the phenomenon of diverse distribution concerning the certain disease. When the distributed condition of imbalanced data is not severe, a good diagnostic model could be obtained using a general analysis method. Otherwise, the proposed method in this study can assist to obtain a more correct diagnostic model. The results showed that our approach achieves a better classification performance than the other methods. Thus our approach can be considered an effective way to enhance the analytical performance for learning imbalanced class distributions. Our plans for future research include exploring how to find better density functions to generate useful synthetic samples to enhance classification performance for specific applications.
